# Overuse of antibiotics for the common cold – attitudes and behaviors among doctors in rural areas of Shandong Province, China

**DOI:** 10.1186/s40360-015-0009-x

**Published:** 2015-03-31

**Authors:** Qiang Sun, Oliver J Dyar, Lingbo Zhao, Göran Tomson, Lennart E Nilsson, Malin Grape, Yanyan Song, Ling Yan, Cecilia Stålsby Lundborg

**Affiliations:** Center for Health Management and Policy, Key Lab of Health Economics and Policy Research of Ministry of Health, Shandong University, 250012 Jinan, Shandong China; Medical Education Centre, North Devon District Hospital, Raleigh Park, Barnstaple, Devon, EX31 4JB UK; Department of Public Health Sciences, Department of Learning, Informatics, Management, Tomtebodavägen 18 A; Medical Management Centre (MMC), Ethics Karolinska Institutet, 171 77 Stockholm, Sweden; Department of Clinical and Experimental Medicine, Clinical Microbiology, Faculty of Health Sciences, Linköping University, 581 85 Linköping, Sweden; Antibiotics and Infection Control Unit, Public Health Agency of Sweden, 17182 Solna, Sweden; School of Public Health, Shandong University, Jinan, Shandong 250012 China; Jinan Central Hospital, Jinan, Shandong 250013 China; Department of Public Health Sciences, Global Health (IHCAR), Tomtebodavägen 18 A, Karolinska Institutet, 1771 77 Stockholm, Sweden

**Keywords:** Antibiotics, Attitudes and behavior, Prescription, Rural area, China

## Abstract

**Background:**

Irrational antibiotic use is common in rural areas of China, despite the growing recognition of the importance of appropriate prescribing to contain antibiotic resistance. The aim of this study was to analyze doctors’ attitudes and prescribing practices related to antibiotics in rural areas of Shandong province, focusing on patients with the common cold.

**Methods:**

A survey was conducted with doctors working at thirty health facilities (village clinics, township health centers and county general hospitals) in three counties within Shandong province. Questions were included on knowledge and attitudes towards antibiotic prescribing. Separately, a random selection of prescriptions for patients with the common cold was collected from the healthcare institutions at which the doctors worked, to investigate actual prescribing behaviors.

**Results:**

A total of 188 doctors completed the survey. Most doctors (83%, 149/180) had attended training on antibiotic use since the beginning of their medical practice as a doctor, irrespective of the academic level of their undergraduate training. Of those that had training, most had attended it within the past three years (97%, 112/116). Very few doctors (2%, 3/187) said they would give antibiotics to a patient with symptoms of a common cold, and the majority (87%, 156/179) would refuse to prescribe an antibiotic even if patients were insistent on getting them. Doctors who had attended training were less likely to give antibiotics in this circumstance (29% vs. 14%, p < 0.001).

A diagnosis of common cold was the only diagnosis reported on 1590 out of 8400 prescriptions. Over half (55%, 869/1590) of them included an antibiotic. Prescriptions from village clinics were more likely to contain an antibiotic than those from other healthcare institutions (71% vs. 44% [township] vs. 47% [county], p < 0.001).

**Conclusions:**

Most doctors have recently attended training on antibiotic use and report they would not prescribe antibiotics for patients with a common cold, even when placed under pressure by patients. However, more than half of the prescriptions from these healthcare institutions for patients with the common cold included an antibiotic. Exploring and addressing gaps between knowledge and practice is critical to improving antibiotic use in rural China.

## Background

Inappropriate antibiotic use is a global problem [1,2]. Several studies in China, the most populated country in the world, have found significant overuse of antibiotics for upper respiratory tract infections [3-5]. Many policies on improving antibiotic use have been issued in China, from the first policy on rational use of antibiotics issued by the Ministry of Health in 1989, to the most strict policy on antibiotic use, launched in 2012. Implementation is lagging behind, however, and irrational use of antibiotic remains common [6].

A detailed context-specific examination of factors which cause high levels of inappropriate antibiotic prescribing is warranted. Few studies to date have been conducted in rural areas of China, although this is where the majority of China’s 1.35 billion inhabitants live [6]. The knowledge, attitudes, and actions of a wide variety of stakeholders including patients, clinicians, and pharmaceutical companies are critical factors that can be modified to improve antibiotic use [7,8]. The present study is part of the ongoing “Sino-Swedish Bilateral Cooperation on Management of Antibiotic Resistance” with the aim of understanding the problems of antibiotic resistance in China and Sweden and developing interventions to address different aspects of the problems. The purpose of this paper is to analyze attitudes and behaviors of doctors at different levels of the rural health care system in relation to antibiotic use, with a focus on patients with the common cold, at health facilities in three counties in Shandong province.

## Methods

### Study sites

**Figure 1:**
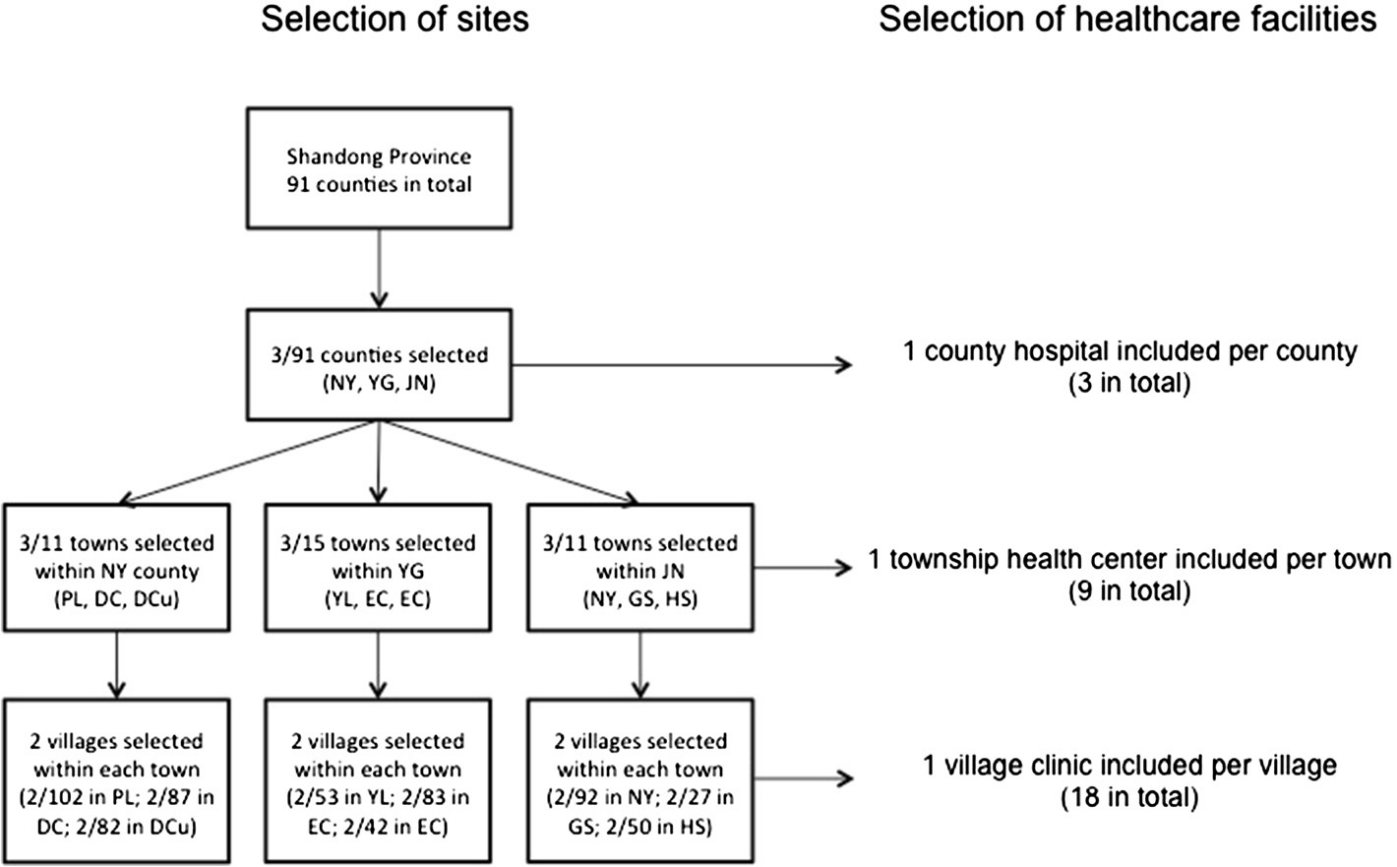
**The selection of study sites and healthcare facilities.**

The study was conducted in 2012 in Shandong Province, located in the eastern part of China. A total of 3 county general hospitals, 9 township health centers and 18 village clinics were selected as study sites using a multistage sampling based on the vertical administrative structure in rural China (see Figure 1). First, three counties (JN, NY and YG) were purposely selected out of a total of 91 counties in Shandong Province, based on geographic location and feasibility of the study. These three counties had around 2.47 million inhabitants in 2012. Secondly, three administrative units were randomly selected in each county. Each administrative unit consists of a town and its surrounding villages. Thirdly, two villages were randomly selected from within each administrative unit. The only county general hospital in each county was included in the study, alongside the only township health centers in each participating administrative unit, and the village clinic within each village.

### Data collection

The data in this paper are from two sources: (i) a survey of doctors and (ii) collection of prescriptions.

#### Survey of doctors

A questionnaire concerning knowledge and attitudes of antibiotic prescribing, particularly in the context of patients with the common cold, was developed jointly between the collaborators in Sweden and China, based on a review of the relevant literature [4,6,8]. It was developed in Chinese and translated into English for the collaborators to discuss. The survey consisted of closed-ended questions with pre-set alternatives. It was tested for language understanding and face validity, and piloted with doctors from the county hospitals and township health centers. The questionnaire was self-completed and paper-based.

The study was limited to clinical doctors who were working with patients, and who had a right to prescribe antibiotics. All doctors working at the selected township health centers and village clinics, and at the county hospitals in the departments of internal medicine, surgery, pediatrics and obstetrics & gynecology, were invited to participate.

#### Collection of prescriptions

Outpatient prescriptions were collected from the selected healthcare institutions in the month prior to the survey (September 2012), in order to analyze actual prescribing behaviors. All available prescriptions were collected from the selected township health centers and village clinics. At the county hospitals, where there was a much higher number of prescriptions than at other institutions, a systematic random sampling methodology was used to generate a maximum of 200 sample prescriptions from each department. The information on the prescriptions included patient name, age, gender, diagnosis, the drug prescribed and medical cost. The name of the prescriber was not collected for individual prescriptions.

For this paper, all prescriptions that included only the single diagnosis of common cold (“Gan Mao”) were analyzed; prescriptions with more than one diagnosis were excluded. No attempt at external validation of diagnosis was made, although it is likely that if the doctor found a more severe disease then they would have written this on the prescription instead.

### Data management and analysis

Data collection was carried out by ten master’s students and researchers from the Center for Health Management and Policy at Shandong University. They were trained by the first author in questionnaire use and the methodology for sampling prescriptions; they also participated in the pilot survey. The first author checked all of the questionnaires on the survey days for quality control of the data collection. Each prescribed antibiotic was coded according to the World Health Organization (WHO) Collaborating Centre for Drug Statistics Methodology, Anatomical Therapeutic Chemical (ATC) classification [9]. All data were entered and validated by two separate data collectors using the EpiData software, and analyzed using STATA 12 software and Microsoft Excel 2010. Categorical data were compared using the Chi-square test, with comparisons made against village clinics unless otherwise stated. The cut-off point for statistical significance was set at p <0.05.

### Ethics Statement

Ethical approval was granted by the ethical committee of the School of Public Health, Shandong University. All of the doctors signed an informed consent form before the start of the questionnaire, and were aware they could withdraw at any point. There was no compensation for participation.

## Results

### Characteristics of the doctors

All eligible doctors working on the day in which the questionnaire was distributed at each facility completed the survey, resulting in a total of 188 completed questionnaires from the different health institutions in the three counties. A summary of the characteristics of the doctors is shown in Table 1. The gender distribution, average age and work experience were broadly similar across all counties. Almost all doctors (99%, 186/187) had a major in Western medicine alone.

**Table 1:** **Characteristics of the doctors completing the questionnaire**

	**Healthcare institution**			**TOTAL**
	**County hospital**	**Township health center**	**Village clinic**	
Total number of doctors	60	98	30	**188**
YG county	20	22	9	**51**
NY county	20	40	11	**71**
JN county	20	36	10	**66**
Male (%)	26 (43)	49 (50)	22 (73)	**97 (52)**
Average age in years	35	36	48	**38**
Average working experience in years	11	14	26	**15**
Doctors with degree of bachelor or above (%)	46 (77)	32 (33)	0 (0)	**78 (41)**

### Attitudes of doctors towards patients with a common cold

The doctors were asked what action they would take when they see a patient with a common cold, with symptoms such as a mild headache, myalgia and malaise. Most doctors (80%, 150/187) suggested that they would encourage the patient to drink water and rest. Many doctors said they would use antipyretics, analgesics or antivirals (67%, 126/187), and only a very small number said they would consider giving an antibiotic (2%, 3/187). Furthermore, the majority of doctors (87%, 156/179) stated they would still refuse to give antibiotics when facing a patient who was insisting on having antibiotics. Doctors were more likely to give antibiotics in this circumstance if they had not attended training (29% vs. 14%, p < 0.001), or worked in a county hospital (23% vs. 12% [township] vs. 13% [village clinic], p < 0.05).

Table 2 shows the results from the questionnaire according to county and healthcare facility type.

**Table 2:** **Attitudes and knowledge of doctors towards antibiotic use and patients with the common cold**

	**YG**			**NY**			**JN**			**Total**		
	**CH**	**THC**	**VC**	**CH**	**THC**	**VC**	**CH**	**THC**	**VC**	**CH**	**THC**	**VC**
No. of doctors	20	22	9	20	40	11	20	36	10	60	98	30
Self-reported behaviour of doctors for patients with symptoms of the common cold:												
Would recommend to drink water and rest (%)	75	73	100	100	74	73	85	89	40	87	79	70
Would give an analgesic, antipyretic or antiviral (%)	55	68	67	30	74	73	80	75	80	55	73	73
Would use antibiotics (%)	0	0	0	5	3	9	0	0	0	2	1	3
Would still refuse to give antibiotics if a patient insisted on receiving antibiotics (%)	80	95	100	100	78	82	50	94	80	77	88	87
General attitudes and knowledege of doctors towards antibiotics:												
Believe that newer antibiotics are more effective (%)	0	0	0	5	0	10	0	6	0	2	2	3
Believe that broader specturm antibiotics are more effective (%)	16	18	22	0	15	40	0	11	0	6	15	21
Are aware that antibiotic treatment guidelines exist at a county or hospital level (%)	100	100	100	100	95	100	100	100	100	100	98	100
Are aware that different levels of doctor have different prescribing rights for antibiotics (%)	100	100	67	100	79	55	95	86	67	98	86	62
Have clinical experience of resistant bacteria (%)	90	59	89	85	44	40	74	92	80	83	66	69
Have participated in training on the use of antibiotics since starting work as a doctor (%)	20	5	0	0	23	18	50	6	20	23	12	13

*Abbreviations*: CH County Hospital, THC Township Health Centre, VC Village Clinic.

### General attitudes and knowledge of doctors towards antibiotics

Most doctors (83%, 149/180) stated that they had participated in some training on antibiotic use since becoming a doctor; doctors in township health centers were less likely to have attended training that doctors at the other institution types (74% vs. 93% [village clinics] vs. 92% [county hospitals], p < 0.001). There was also some variation between the three counties, with training attendance rates highest amongst doctors from JN (93% vs. 80% [YG] and 74% [NY]). Doctors with a bachelor’s degree were as likely to have attended training as those without a degree. Of the doctors who provided dates for the training, 97% (112/116) had had training within the three years prior to the survey.

Almost all doctors (98%, 182/186) did not think that newer antibiotics are more effective, and most doctors (87%, 156/179) did not think that antibiotics having a broader antimicrobial spectrum imply a better effect. There was no difference in these responses if the doctors had received training, nor if they had a bachelor’s degree. Doctors in the county hospitals were less likely to think that broader spectrum antibiotics had better effects (6% vs. 15% [township center] and 21% [village clinic], p < 0.01).

The vast majority of doctors (99%, 182/184) were aware that antibiotic guidelines exist either at a county or hospital level. Most doctors (86%, 160/185) were aware that different levels of doctor have different rights to prescribe antibiotics (for instance, prescription of some antibiotics is restricted to certain specialists). Village clinic doctors were more likely to be unaware of the different prescribing rights compared with doctors in the other healthcare institutions (17% vs. 2% [township center doctors] and 0% [county hospital doctors], p < 0.001); there was no variation based on training received.

A quarter of doctors (27%, 51/188) said that they had not had clinical experiences with antibiotic-resistant bacteria. Doctors were more likely to say they had encountered resistant bacteria if they had a bachelor’s degree (81% vs. 65%, p < 0.001), had attended training on antibiotic use (79% vs. 47%, p < 0.001), or worked at a county hospital (83% vs. 66% [township] vs. 69% [village], p < 0.001).

### Analysis of prescriptions

A total of 8400 prescriptions were analyzed from the healthcare institutions. Of these, 1590 (19%) cited a single diagnosis of the common cold, with over half (55%, 869/1590) of these prescriptions including an antibiotic (Table 3). Common cold prescriptions from village clinics were more likely to contain a prescription for an antibiotic than prescriptions from other institutions (71% vs. 44% [township] vs. 47% [county], p < 0.001).

**Table 3:** **Common cold prescriptions: amounts and classes of antibiotics**

	**Healthcare institution**			
	**County hospital**	**Township health center**	**Village clinic**	**TOTAL**
Number of healthcare institutions	3	6	18	**27**
Total number of prescriptions	1303	4799	2298	**8400**
Total number of common cold prescriptions	122	839	629	**1590**
Number of cold prescriptions with antibiotic (%)	57 (47)	366 (44)	446 (71)	**869 (55)**
Mean number of antibiotics prescribed	1.01	1.17	1.11	**1.12**
**Amount of types of antibiotics prescribed for the diagnosis of common cold**				
Other beta-lactam antibacterials (J01D)	22	156	210	**388**
Macrolides, lincosamides and streptogramins (J01F)	31	158	145	**334**
Beta-lactam antibacterials, penicillins (J01C)	3	76	77	**156**
Quinolone antibacterials (J01M)	1	14	30	**45**
Other antibacterials (J01X)	1	18	18	**37**
Sulfonamides and trimethoprim (J01E)	0	3	6	**9**
Aminoglycoside antibacterials (J01G)	0	1	7	**8**
Tetracyclines (J01A)	0	1	0	**1**

A total of 979 antibiotics were prescribed, with a mean of 1.12 antibiotics per prescription. At county hospitals, five classes of antibiotics were prescribed, compared with eight in the township health centers, and seven in the village clinics. The most frequently used classes of antibiotics across all healthcare institutions were ‘other beta-lactam antibacterials’ (J01D), including cephalosporins and carbapenems; ‘macrolides, lincosamides and streptogramins’ (J01F); and ‘beta-lactam antibacterials, and penicillins’ (J01C) (Table 3).

## Discussion

To date, few studies have investigated the attitudes and practices of doctors concerning antibiotic use in rural China [6,10]. In our study we have combined analysis of prescription data and doctors’ attitudes and knowledge on antibiotics from three different levels of rural healthcare institutions, within three counties in Shandong province. Although almost all doctors stated they would not use antibiotics for a patient with a common cold in our questionnaire, we found that at least one antibiotic was present on over half of all prescriptions for patients with a common cold taken from the institutions these doctors work at. Gaps between reported knowledge and actual practice within antibiotic prescribing are commonly encountered. This high prescription rate of antibiotics in the context of viral upper respiratory tract infections is in line with the results of a recent systematic review suggesting that almost half of all outpatient appointments in China result in a prescription for an antibiotic [6]. At the time of the current study, doctors were able to make a profit from individual drug prescriptions, including antibiotics, and this may have stimulated over-prescribing of antibiotics [11-13]. This is particularly important at the current stage of comprehensive health system reform occurring now in China.

Reynolds *et al.* carried out semi-structured interviews with doctors from a variety of healthcare institutions in Guizhou province in southern China [14]. Their findings suggest that although doctors are aware that antibiotics are not needed to treat the common cold, antibiotics are often given under the belief that they might speed recovery, and also in response to patient expectations. A recent study of caregivers in rural China [15] found that 80% of parents thoughts that antibiotics help with viral infections. Undergraduate education and postgraduate training of doctors can help address such misconceptions, and provide strategies to respond to patient expectations [16,17]. A high proportion of doctors in our study have attended training on antibiotics since qualifying, and for the vast majority this training has occurred recently. It is unclear why doctors from township health centers were less likely to have attended such training than village doctors or doctors from county hospitals. It may be that doctors from township health centers have received less encouragement or have fewer opportunities to attend training than doctors from other health facility levels.

Our study has assessed attitudes and practice across three rural counties and three levels of the healthcare system, with a high response rate. It has, however, several important limitations. Firstly, the questionnaire was self-completed and consequently some individuals may have modified their answers to meet social expectations. Secondly, it was not possible to validate the diagnosis of common cold on the prescriptions, nor was data available on clinical outcomes. Thirdly, it is possible that some patients attended the healthcare institutions and received a diagnosis of common cold, but did not receive a prescription; however, all patients will generally get a prescription at these healthcare facilities. Fourthly, although the prescriptions analyzed are all from healthcare institutions where the survey respondents worked, we are unable to guarantee that the doctors who responded to the survey are responsible for all of the prescriptions analyzed. In future studies researchers should consider collecting the name of the prescriber from each prescription, enabling further investigation of the relationship between self-reported knowledge and attitudes and actual practice at the level of the individual.

## Conclusions

This study showed a substantial gap between rural doctors’ attitudes and practice regarding antibiotic prescribing. Attitudes were in line with recommendations, whereas practice showed a high level of inappropriate prescribing of antibiotics for the common cold. Exploring and addressing gaps between knowledge and practice is critical to improving antibiotic use in rural China.
